# 吉西他滨调整方案治疗晚期非小细胞肺癌的Ⅱ期临床试验

**DOI:** 10.3779/j.issn.1009-3419.2012.01.01

**Published:** 2012-01-20

**Authors:** 璐璐 缪, 云 范, 志煜 黄, 能明 林, 吕宏 罗, 海峰 余

**Affiliations:** 310022 杭州，浙江省肿瘤医院化疗中心 Chemotherapy Center, Zhejiang Cancer Hospital, Hangzhou 310022, China

**Keywords:** 肺肿瘤, 吉西他滨, 联合方案, Lung neoplasms, Gemcitabine, Combined chemotherapy regimens

## Abstract

**背景与目的:**

吉西他滨与铂类的联合化疗是晚期非小细胞肺癌（non-small cell lung cancer, NSCLC）最常用的治疗方案。通常3周方案中的吉西他滨需间隔1周给药。为提高依从性，本研究将吉西他滨第8天给药时间调整为第5天，并评价调整方案一线治疗晚期NSCLC的疗效及安全性。

**方法:**

2007年10月-2009年10月共入组83例晚期NSCLC患者，采用吉西他滨1, 000 mg/m^2^-1, 250 mg/m^2^第1天、第5天静脉滴注30 min，联合顺铂75 mg/m^2^，或联合卡铂（AUC=5）第1天静滴，每21天为1周期，每例至少完成2周期治疗后评价疗效，观察毒性反应及无进展生存期和总生存期。

**结果:**

83例患者的客观有效率为37.3%，中位无进展生存期和中位生存期分别为6.1个月和15.0个月，1年、2年生存率分别为57.8%与16.2%。调整方案的主要不良反应为血液学毒性与胃肠道反应，Ⅲ度-Ⅳ度白细胞、血红蛋白、血小板减少发生率分别为26.5%、10.8%、7.2%，联合顺铂治疗组Ⅲ度-Ⅳ度胃肠道反应发生率为27.5%。无治疗相关死亡。

**结论:**

吉西他滨联合铂类5天调整方案一线治疗晚期NSCLC疗效肯定，毒副反应可耐受，值得进一步开展随机对照研究。

## Abstract (en)

**Background and objective:**

Gemcitabine-platinum-combined with chemotherapy is the most common treatment for advanced non-small cell lung cancer (NSCLC). Gemcitabine is administered once a week in a general three-week schedule. In the present study, gemcitabine is administered on d1 and d5 to improve compliance, and the efficacy and safety of the improved regimen is evaluated in untreated patients with advanced NSCLC.

**Methods:**

A total of 83 patients were enrolled between October 2007 and October 2009. In each cycle, gemcitabine was administered at a dose of 1, 000 mg/m^2^-1, 250 mg/m^2^ via a 30 min intravenous infusion on d1 and d5 followed by cisplatin at a dose of 75 mg/m^2^ or carboplatin (AUC=5) on d1 every three weeks. At least two cycles of chemotherapy were completed in each case, and clinical response and toxicity of the regimen were observed.

**Results:**

The objective response rate was 37.3%. The median progression free survival and overall survival time were 6.1 months and 15.0 months, respectively. The one-year and two-year survival rates were 57.8% and 16.2%, respectively. Myelosuppression and gastrointestinal responses were the main toxicities. The incidence of grade 3/4 of leucopenia, hypohemia, and thrombocytopenia were 26.5%, 10.8% and 7.2%, respectively. A total of 27.5% of the patients in the cisplatin group had grade 3/4 gastrointestinal responses. Treatment related deaths were not observed in this study.

**Conclusion:**

The regimen is active and well-tolerated in untreated patients with advanced NSCLC. Further randomized controlled studies are necessary.

吉西他滨是一种新型抗代谢药物，化学名为2′-脱氧-2′, 2′-盐酸双氟胞苷（dFdC），是一种具有广谱抗实体瘤活性的脱氧胞苷类似物。自1996年被美国食品药品监督管理局批准美国上市治疗进展期及转移性非小细胞肺癌（non-small cell lung cancer, NSCLC）以来，在临床上该药已被广泛应用及研究，吉西他滨联合铂类方案为目前治疗晚期NSCLC最常用的一线化疗方案^[1-3]^。吉西他滨与铂类的两药联合方案治疗NSCLC有效率为28%-54%，毒副反应可耐受^[4, 5]^。在常用的3周方案中，吉西他滨在第1天、第8天应用。为提高患者依从性，本研究尝试将第8天应用吉西他滨调整为第5天给药。调整方案不仅可以缩短住院治疗时间，还可避免大多数患者因第8天血象降低而使吉西他滨给药推迟。2007年10月-2009年10月共入组83例未经治疗的晚期NSCLC患者进行Ⅱ期临床研究，本文旨在对吉西他滨联合铂类调整方案治疗的安全性和疗效进行初步评价。

## 1 资料与方法

### 1.1 研究对象和纳入标准

经组织学或细胞学确诊的不能手术的初治晚期（Ⅲb期或Ⅳ期）NSCLC，包括手术后复发患者，至少具有一个可测量的病灶。其它入选标准包括：①未经化疗或放疗；②全身功能状态评分（performance status, PS）为0分-2分；③年龄 > 18岁，≤75岁；④预计生存期≥3个月；⑤骨髓造血功能基本正常：外周血白细胞计数≥3.5×10^9^/L，中性粒细胞绝对值计数≥1.5×10^9^/L，血红蛋白≥9.0 g/L，血小板计数≥100×10^9^/L；⑥肝肾功能：血清转氨酶≤正常值上限的2倍，总胆红素≤正常值上限的1.5倍，血清肌苷≤正常值上限的1.5倍或血清肌苷清除率≥50 mL/mim。排除标准：①妊娠期及哺乳期妇女；②有症状的脑转移；③有其它恶性肿瘤病史，除外皮肤基底细胞癌和子宫颈原位癌；④有严重感染或器质性疾病的患者。

### 1.2 治疗计划

采用吉西他滨联合顺铂（gemcitabine+cisplatin, GP）方案或吉西他滨联合卡铂（gemcitabine+carboplatin, GC）方案。吉西他滨用法为1, 000 mg/m^2^-1, 250 mg/m^2^，30 min内静脉滴注，第1、第5天各1次；顺铂用法总剂量为75 mg/m^2^，第1天；或卡铂，剂量为曲线下面积（area under the curve, AUC）=5 mg（min·mL^-1^），第1天静脉滴注。每3周重复，每2个周期评价疗效，每例患者至少进行2个周期化疗，最多不超过6个周期。化疗前常规使用5-羟色胺拮抗剂以预防呕吐。剂量调整计划：每周期治疗开始前，中性粒细胞绝对值计数需≥1.5×10^9^/L，血小板计数≥100×10^9^/L，无Ⅲ度和Ⅳ度的非血液学毒性，如果未达到上述标准，下一疗程化疗延迟，直至达到上述标准；如果延迟治疗超过14天仍未达到标准，患者将退出研究。疗程第1天化疗剂量调整：如前面治疗过程中出现粒细胞减少性发热或Ⅳ度血小板减少，或出血性的Ⅱ度、Ⅲ度血小板减少，吉西他滨剂量调整为原剂量的75%-90%；顺铂剂量调整为原剂量的90%；第5天的吉西他滨剂量调整方案：如中性粒细胞绝对值计数＜（0.5-0.99）×10^9^/L或血小板计数＜（50-99）×10^9^/L，吉西他滨剂量调整为原剂量的75%-90%。如患者需要第3次的剂量调整，或有大于Ⅲ度的非血液学毒性（除外呕吐、疲劳或可逆转的转氨酶升高）将退出研究。

### 1.3 安全性与疗效评价方法

化疗期间每周检查血常规2次，至化疗结束后3周止。每疗程化疗前均常规检查血肝肾功能及电解质。化疗期间记录各种不良事件的发生及处理，不良反应分级依据《WHO抗癌药物急性及亚急性毒副反应分级标准》评定，分为0级-Ⅳ级。疗效评价依据实体肿瘤疗效评价标准(RECIST 1.1版），分为完全缓解（complete response, CR）、部分缓解（partial response, PR）、疾病稳定（stable disease, SD）和疾病进展（progressive disease, PD）。

### 1.4 随访方式

采用信访或电话随访，随访截止时间为2011年4月，生存时间计算方法为从化疗开始至死亡或末次随访时间。

### 1.5 统计学方法

采用SPSS 16.0软件进行统计学分析，率的比较采用χ^2^检验，*Kaplan*-*Meier*法计算生存率，*Log*-*rank*检验比较生存率差异。*P*＜0.05为差异有统计学意义。

## 2 结果

### 2.1 临床资料

2007年10月-2009年10月共入组83例患者，包括男性49例，女性34例。年龄35岁-75岁，中位年龄为56岁；PS 0分-1分70例，2分13例；腺癌44例，鳞癌21例，腺鳞癌1例，其它病理类型17例；Ⅲb期（依据第七版AJCC肺癌TNM分期标准）30例，Ⅳ期53例。治疗完成情况：83例患者中采用吉西他滨联合顺铂方案化疗者69例，联合卡铂者14例，共完成346个周期化疗，平均每例患者完成4.17个周期。30例Ⅲb期患者中有21例进行常规胸部放疗。

### 2.2 近期疗效评价

全组患者中CR 1例，PR 30例，客观有效率为（objective response rate, ORR）为37.3%。卡铂组与顺铂组的ORR分别为28.6%（4/14）和39.1%（27/69），两组差异无统计学意义（χ^2^=1.95, *P*=0.66）。

### 2.3 生存情况

全组患者无进展生存期（progression free survival, PFS）为6.1个月，中位生存期（overall survival, OS）为15.0个月（图 1A），1年与2年生存率分别为57.8%与16.2%。GC组与GP组中位生存时间分别为13.0个月和16.0个月（*P*=0.79），两组相比差异无统计学意义（图 1B）。

**1:**
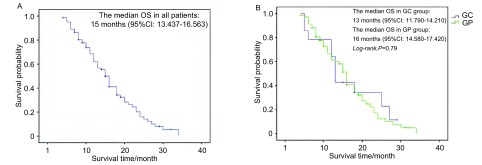
83例患者总的生存曲线（A）和GP组与GC组的生存期比较（B） The survival curve of all 83 patients(A)and the comparison of overall survival(OS)in GC and GP groups(B) GP: gemcitabine+cisplatin; GC: gemcitabine+carboplatin.

### 2.4 不良反应

主要的不良反应为骨髓抑制。Ⅲ度-Ⅳ度白细胞、血红蛋白、血小板减少发生率分别为26.5%、10.8%、7.2%。13例患者出现中性粒细胞减少性发热，其中7例出现感染，经粒细胞集落刺激因子（G-CSF）及抗炎治疗后感染控制，无1例发生败血症、感染性休克等严重并发症。治疗期间10例贫血患者需要输注红细胞以改善症状，3例患者输注了血小板预防出血，均未出现皮肤、粘膜出血及内脏出血。全组患者中6例因血液学毒性调整剂量。非血液学毒性中胃肠道反应最为明显，顺铂治疗组Ⅲ度-Ⅳ度胃肠道反应为19例（27.5%），明显高于卡铂组1例（7.1%）。其它不良反应有转氨酶升高、皮疹、肾功能损害、外周神经毒性，大多数为轻度不良反应，对症治疗后恢复正常。全组患者无治疗相关死亡。

## 3 讨论

虽然靶向治疗在表皮生长因子受体（epidermal growth factor receptor, EGFR）基因突变的晚期NSCLC患者的一线治疗中取得了明确的疗效，但对于大多数晚期NSCLC患者取得组织学标本进行基因突变检测仍然受限。TORCH研究^[6]^的结论指出，对于*EGFR*基因突变状态未知的NSCLC患者，一线接受化疗患者的生存优于一线接受靶向治疗患者。而吉西他滨联合铂类是晚期NSCLC一线治疗最常用的化疗方案^[1-3]^。吉西他滨与铂类的作用机制不同，在疗效上有协同作用^[7, 8]^，而主要毒性作用无明显叠加，两者联合治疗晚期NSCLC疗效已被国外30多项Ⅲ期临床试验所评估^[9]^。ECOG 1594研究比较了4个常用的标准一线化疗方案的疗效，在生存期和有效率方面4个方案相似，而吉西他滨联合顺铂在疾病进展时间（time to progression, TTP）和1年生存率方面占有优势^[10]^。

吉西他滨联合铂类的最佳方案尚无统一，在早期关于吉西他滨的Ⅰ期临床研究中，常用的治疗方案是间隔1周给药^[11]^，更短的给药间隔被认为与血小板减少及发热、皮疹、低血压等特殊的不良反应有关。因此随后进行的Ⅱ期临床研究重点在选择3周或4周方案及确定剂量、是否与顺铂联合等问题上^[12, 13]^。最近也有部分学者重新对吉西他滨的给药间隔时间进行了相关研究。为提高剂量强度，López等^[14]^每2周给予吉西他滨2, 500 mg/m^2^，每28天为1周期，共49例Ⅲb期及Ⅳ期患者的客观有效率为38.4%，但有1例治疗相关死亡，2例出现严重血管毒性。Parra等^[13]^将吉西他滨在每21天的第1、4天应用，观察到在39例患者（34例NSCLC和5例上皮性肿瘤）中联合方案可达到有效的剂量强度，认为副反应与每周方案相似。

根据药代动力学，吉西他滨（dFdC）进入体内后需要在细胞内通过脱氧胞嘧啶核苷激酶磷酸化成为活性形式的二磷酸及三磷酸双氟胞苷（dFdCDP和dFdCTP），才能抑制DNA合成从而发挥抗肿瘤作用。30 min静脉滴注后dFdC半衰期仅为11 min-30 min，很快转变成无活性的代谢产物2, 2’-双氟脱氧胞嘧啶核苷（dFdU），而dFdU的半衰期在8 h-84 h不等。有研究^[15]^报道长时间暴露于dFdU可影响吉西他滨的代谢过程而增加其相关毒性。本研究组前期进行的吉西他滨调整方案的药代动力学研究^[16]^发现，第5天给药的药代动力学参数无统计学差异，第5天给药前吉西他滨代谢产物（dFdU）仅有微量残留。这为吉西他滨的第1、5天给药提供了药理学上的支持。

本研究吉西他滨联合铂类5天调整方案的主要不良反应为骨髓抑制。22例患者（26.5%）出现Ⅲ度-Ⅳ度的白细胞减少，其中7例出现感染。9例（10.8%）出现Ⅲ度-Ⅳ度贫血，6例（7.25%）出现Ⅲ度-Ⅳ度血小板减少。10例患者接受了红细胞输注以改善症状，3例患者输注血小板预防出血。6例因血液学毒性而调整剂量，无治疗相关死亡。与其它研究中每周给药的标准方案^[4, 5, 17-19]^相比，血液学毒性未明显增加。其它反应如转氨酶升高、皮疹、肾功能损害、外周神经毒性等多为轻度，均可耐受。

本研究共入组83例患者，其中吉西他滨联合顺铂69例，联合卡铂14例。因考虑研究重点为吉西他滨调整用药后对不良作用及疗效的影响，未对卡铂与顺铂两组进行随机分组。根据国外文献报道多选用吉西他滨联合顺铂方案化疗，故本研究对PS评分能耐受的患者尽量选择顺铂化疗，因而造成两组例数差异较大。Ⅲ度-Ⅳ度胃肠道反应发生率顺铂治疗组（27.5%）明显高于卡铂组（7.1%），可能也与组间例数差异有关。

文献^[4, 5, 17-19]^报道吉西他滨每周给药的联合方案治疗晚期NSCLC的有效率为30%-59%，中位生存时间为8.4个月-15.4个月（表 1）。本研究显示吉西他滨联合铂类5天调整方案治疗晚期NSCLC客观有效率为37.3%，中位PFS为6.1个月，中位生存时间为15.0个月。同时我们前期进行的Ⅱ期药代动力学研究^[16]^显示，28例晚期NSCLC患者采用5天调整方案的总缓解率为33.3%，中位生存时间为13个月，均显示调整方案与每周治疗方案疗效接近。

**1:** 吉西他滨联合铂类方案的临床研究 Studies of gemcitabine-platinum combined chemotherapy

Author/reference	Weeks	Schedule and dose	Hematologic toxicity	Median suvival (month)	Response rate (%)
Manegold, *et al*^[4]^ (2000)	3	G (1, 000-1, 250 mg/m^2^) d1, 8 C (75 mg/m^2^) d1	-	8.4-15.4	30-59
Sandler, *et al*^[5]^ (2000)	4	G (1, 000 mg/m^2^) d1, 8, 15 C (100 mg/m^2^) d1	Neutropaenia at grade 4 (35.3%) Thrombocytopaenia at grade 4 (25.4%)	9.1	30.4
Scagliotti, *et al*^[17]^(2002)	3	G (1, 250 mg/m^2^) d1, 8 C (75 mg/m^2^), d2	Neutropaenia (17%) Thrombocytopaenia (16%)	9.8	30
Jassem, *et al* ^[18]^ (2002)	4	G (1, 000 mg/m^2^) d1, 8, 15 C (100 mg/m^2^) d2	Anemia at grade 3/4 (30%) Neutropaenia at grade 3/4 (58%) Thrombocytopaenia at grade 3/4 (65%)	11.0	41
Gaafar, *et al*^[19]^ (2004)	3/4	G (1, 000-2, 500 mg/m^2^) d1, 8, (15) C (80-100 mg/m^2^), d1/15	Anemia at grade 3/4 (18.6%) Neutropaenia at grade 3/4 (32.6%) Thrombocytopaenia at grade 3/4 (20.4%)	9	41.7
G: gemcitabine; C: cisplatin.					

总之，吉西他滨联合铂类5天调整方案是一种新的尝试，研究初步证实了调整方案一线治疗晚期NSCLC疗效肯定、耐受性较好，值得进一步开展与标准方案进行对比的随机对照研究。

## References

[1] Rinaldi M, Cauchi C, Gridelli C (2006). First line chemotherapy in advanced or metastatic NSCLC. Ann Oncol.

[2] Gridelli C, Ardizzoni A, Barni S (2011). Medical treatment choices for patients affected by advanced NSCLC in routine clinical practice:Results from the Italian observational "SUN" (Survey on the IUng cancer maNagement) study. Lung Cancer.

[3] Ramalingam S, Belani C (2008). Systemic chemotherapy for advance non-small cell lung cancer: recent advances and future directions. Oncologist.

[4] Manegold C, Zatloukal P, Krejcy K (2000). Gemcitabine in non-small cell lung cancer (NSCLC). Invest New Drugs.

[5] Sandler AB, Nemunaitis J, Denham C (2000). Phase Ⅲ trial of gemcitabine plus cisplatin versus cisplatin alone in patients with locally advanced or metastatic non-small-cell lung cancer. J Clin Oncol.

[6] Gridelli C, Butts C, Ciardiello F (2008). An international, multicenter, randomized phase Ⅲ study of first-line erlotinib followed by second-line cisplatin/gemcitabine versus first-line cisplatin/gemcitabine followed by second-line erlotinib in advanced non-small-cell lung cancer: treatment rationale and protocol dynamics of the TORCH trial. Clin Lung Cancer.

[7] Bergman AM, Ruiz van Haperen VW, Veerman G (1996). Synergistic interaction between cisplatin and gemcitabine *in vitro*. Clin Cancer Res.

[8] Peters GJ, Bergman AM, Ruiz van Haperen VW (1995). Interaction between cisplatin and gemcitabine *in vitro* and *in vivo*. Semin Oncol.

[9] Wang L, Liao ML, Li LY (2004). Clinical benefit of gemcitabine plus cisplatin 3-week regimen for patients with advanced non-small-cell lung cancer (NSCLC): a prospective observational study. Chin Med J (Engl).

[10] Schiller JH, Harrington D, Belani CP (2002). Comparison of four chemotherapy regimens for advanced non-small-cell lung cancer. N Eng J Med.

[11] Abbruzzese JL, Grunewald R, Weeks EA (1991). A phase Ⅰ clinical, plasma and cellular pharmacology study of gemcitabine. J Clin Oncol.

[12] Anderson H, Lund B, Bach F (1994). Single-agent activity of weekly gemcitabine in advanced non-small-cell lung cancer: a phase Ⅱ study. J Clin Oncol.

[13] Parra HS, Cavina R, Latteri F (2007). Cisplatin plus gemcitabine on days 1 and 4 every 21 days for solid tumors: result of a dose-intensity study. Invest New Drugs.

[14] López-Vivanco G, Viteri A, Barceló R (2005). Biweekly administration of cisplatin/gemcitabine in advanced non small cell lung cancer. Am J Clin Oncol.

[15] Veltkamp SA, Pluim D, van Eijndhoven MA (2008). New insights into the pharmacology and cytotoxicity of gemcitabine and 2', 2'-difluorodeoxyuridine. Mol cancer Ther.

[16] Fan Y, Lin NM, Ma SL (2010). Phase Ⅱ trial of gemcitabine plus cisplatin in patients with advanced non-small cell lung cancer. Acta Pharmacol Sin.

[17] Scagliotti GV, De Marinis F, Rinaldi M (2002). Phase Ⅲ randomized trial comparing three platinum-based doublets in advanced non-small-cell lung cancer. J Clin Oncol.

[18] Jassem J, Krzakowski M, Roszkowski K (2002). A phase Ⅱ study of gemcitabine plus cisplatin in patients with advanced non-small cell lung cancer: clinical outcomes and quality of life. Lung cancer.

[19] Gaafar RM, Hamza R, Khaled HM (2004). Gemcitabine and cisplatin in the treatment of advanced non-small cell lung cancer: National Cancer Institute Cairo experience. J Egypt Natl Canc Inst.

